# Enthesitis in Bowel-Associated Dermatosis-Arthritis Syndrome in an Ulcerative Colitis Patient

**DOI:** 10.1155/2022/4556250

**Published:** 2022-04-12

**Authors:** Khalid S. Alshahrani, Abdulellah M. Almohaya, Rayan S. Hussein, Rania H. Ali

**Affiliations:** ^1^Internal Medicine Department, Ad-Diriyah Hospital, Ministry of Health, Riyadh, Saudi Arabia; ^2^Department of Medicine, University of Medical Sciences and Technology, Khartoum, Sudan; ^3^Department of Medicine, Alzaiem Alazhari University, Khartoum, Sudan

## Abstract

Bowel-associated dermatosis-arthritis syndrome (BADAS) is a rare and recurrent neutrophilic dermatosis condition. Due to the rarity of this syndrome and the difficulty of the disease recognition and management, there was no clear reported incidence rate of this syndrome. 20% of patients after ileojejunal bypass surgery for morbid obesity were reported, by Jorizzo et al., to have BADAS. The underlying etiology of BADAS is not fully understood; therefore, the diagnosis of this condition is difficult and the approach for the management as well. Herein, we report a case of BADAS with unusual musculoskeletal presentation.

## 1. Introduction

The presentation of BADAS commonly consists of skin eruptions, fever, malaise, arthralgia, and myalgias. It was first described as a consequence of gastric bypass surgery; It has been reported to be associated with inflammatory bowel disease (IBD) and peptic ulcer disease [1]. Histologically, neutrophil infiltration and dermal vascular changes were observed, suggesting that immune complex-mediated processes are critical for disease pathophysiology [2]. We report a case of ulcerative colitis complicated by BADAS with enthesitis as the main symptom. To our knowledge, this is the first case reported in the literature in which musculoskeletal manifestations, especially enthesitis, were the predominant presentation.

## 2. Case Presentation

A 39-year-old gentleman, with a history of ulcerative colitis, presented to our institute's emergency department with a 4-day history of bilateral severe pain and swelling at the insertion sites of Achilles' tendons, as well as bilateral knee swelling and tenderness. There was a history of persistent fever (>38.7°C) that poorly respond to nonsteroidal anti-inflammatory drugs. A new skin rash involving the upper and lower limbs was noticed by the patient 2 weeks prior to his admission. The ulcerative colitis (US) diagnosis was made almost 3 years before the current presentation. The diagnosis of UC disease was based on the clinical history of bloody diarrhea, loss of weight, and the histopathological evidence of typical architecturally distorted and inflamed crypt (cryptitis) with inflammatory cells in the lamina propria at the biopsy taken during the colonoscopy. At the time of diagnosis of the UC disease, the patient received an induction treatment with methylprednisolone that was followed by a maintenance regimen that included a course of prednisone with a tapering plan, mesalamine (5-aminosalicylic acid), and azathioprine. Nevertheless, he never had a complete disease remission. However, he was continued on the maintenance therapy until current presentation. He denied any history of unprotected sexual intercourse, recreational drug abuse, recent travel, or any history of recently initiated medications.

**Figure 1:**
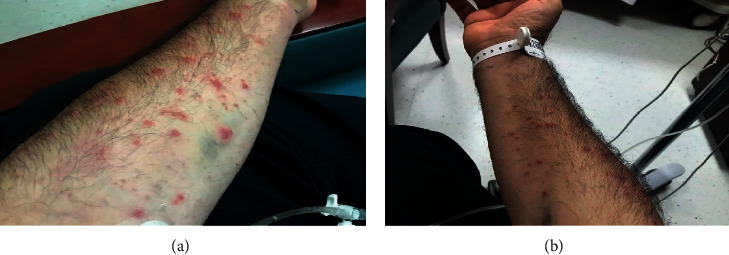
Skin lesions (papular, maculopapular, and urticaria-like rash) involving left (a) and right (b) forearms in a 39-year-old gentleman with bowel-associated dermatosis-arthritis syndrome (photo taken with written permission).

During the initial assessment, the patient was found febrile (38.5°C) with tachycardia of 117 beats/minute. Cutaneous examination showed erythematous papular, maculopapular, and urticaria-like rash with some vesiculopustular lesions, some of which were already crusted. The lesions had an average diameter of 3–7 mm that was not well demarcated and were distributed predominantly on the limbs rather than the trunk. There were no exudates (Figures 1(a) and 1(b)). Joint examination revealed severe tenderness over the insertion of the Achilles' tendons bilaterally with significant swelling, redness, and restriction of movement of the ankle joints. Swelling of both the knees, erythema, and tenderness were noticed while examining the knees. Otherwise, the clinical examination was not revealing. Colour Doppler ultrasound had confirmed the diagnosis of enthesitis and enthesopathy at the insertion sites of Achilles' tendons.

His initial laboratory investigations demonstrated an elevated erythrocyte sedimentation rate (ESR = 84 mm/h, reference range 0–21 mm/h) and C-reactive protein (CRP = 64 mg/L, reference range 0–8 mg/L). Basic investigations were essentially normal, as well as the blood culture which was reported negative after 5 days of incubation. Further autoimmune workup, including the rheumatoid factor, antinuclear antibody, double-stranded DNA antibody, complement component (C3 and C4), cytoplasmic and perinuclear antineutrophil cytoplasmic antibodies, HIV, and hepatitis B and C workup were all reported normal (Table 1). A colonoscopy was done, which revealed only hyperemia with mild edematous mucosal changes in the sigmoid colon with no active ulcerations or deep inflammation in mucosa.

**Figure 2:**
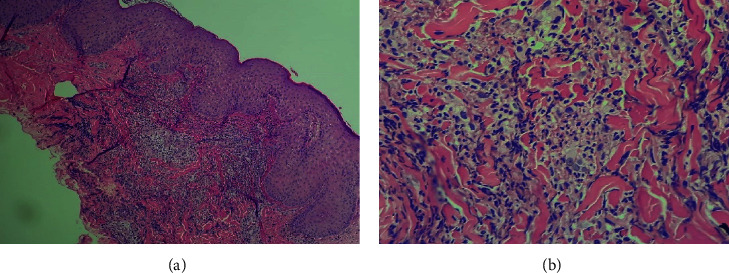
Dermal pathology showing lymphohistiocytic infiltrate and neutrophil granulocytes, consistent with bowel-associated dermatosis-arthritis syndrome (BADAS). Power magnification 40x (a) and 200x (b) of skin slides (hematoxylin-eosin staining).

A bedside skin biopsy done from the left upper arm skin rash demonstrated neutrophilic, perivascular lymphohistiocytosis infiltrates with edema in the dermis (Figures 2(a) and 2(b)). The findings are consistent with neutrophilic dermatosis, which is along with the history of the UC disease, and the current clinical presentation led us to the diagnosis of “BADAS.”

Thereafter, the patient was initiated on metronidazole and fluoroquinolone, followed by a course of tapering dose of oral prednisolone of 0.5 mg/kg. In four weeks of follow-up, the patient showed almost complete recovery. The rash resolved as well as the swelling and the pain of the knee joints by mid of the third week. There was a significant improvement of the Achilles' tendon enthesitis including almost complete reduction of the previously noticed swelling, no more tenderness, and no restriction of movement of the ankle joints. Furthermore, upon admission, the patient was having a history of recurrent diarrhea with mild abdominal pain but no bleeding per rectum. These symptoms, although, improved after initiating the treatment but did not disappear completely. For that reason, the gastrointestinal team was involved in the patient's care for better control of the UC disease and long-term disease stability.

## 3. Discussion

BADAS manifestations can begin with a flu-like picture followed by skin lesions and various other signs and symptoms. The cutaneous manifestations of BADAS may be masked at early presentation and then progress to painful, itchy erythematous plaques, mainly but not limited to the upper extremities and trunk. In most cases, the lesions transform into purpuric papules or papulopustular pustules [1, 2]. In our case, however, the atypical presentation of the rash was not painful, was not primarily pruritic, and involved the extremities rather than the trunk, making the diagnosis more challenging. This underscores the importance of considering the diagnosis of BADAS in any patients with inflammatory bowel disease presenting with atypical mucocutaneous presentation and various acute musculoskeletal manifestations.

Currently, little is known about the pathogenesis of BADAS. It has been suggested that intestinal inflammation and bacterial overgrowth may lead to abnormal activation of the immune system, which in turn leads to the deposition of immune complexes on the skin and synovium [3]. The histological appearance of BADAS is not characteristic. Sweet syndrome is histologically identical to BADAS, and the clinical presentation should guide the physician in making the correct diagnosis. Histological biopsy findings of bowel-associated dermato-arthritis syndrome are characterized by predominantly neutrophilic infiltration and lack of fibrinoid necrosis [4]. The bowel-associated dermatosis-arthritis syndrome was reported in both ulcerative colitis and Crohn's diseases in the literature with almost the same extraintestinal presentation. Yet, there was no clear reported predominance of one disease over the other concerning the association with BADAS.

Currently, there is no consensus on the recommended treatment for BADAS. Focusing on treating the underlying disease (i.e., IBD) is essential to control the manifestation of BADAS. In case of bacterial overgrowth or an infectious cause, antibiotics are most frequently used. For patients with an underlying inflammatory bowel disease, controlling disease activity helps to control BADAS. Regaining normal bowel anatomy in bypass surgeries complicated with BADAS has been effective in many cases [5]. The decision to consider the treatment with antibiotics in addition to steroids in this patient was guided by the indeterminate cause of the case since fever and the elevated inflammatory markers can present in inflammation and infectious etiologies. Although BADAS is often overlooked when compiling differential diagnoses for neutrophilic dermatoses, it should be considered in patients with bowel disease or the recent history of bowel surgery. Because this syndrome can recur, early diagnosis can help prevent the recurrence of BADAS [6]. Proper identification and control of BADAS precipitating factors and underlying disease is critical. In our patients, UC disease was not effectively controlled for a long period of time. Recently, successful treatment of BADAS with the interleukin-12 inhibitor ustekinumab has been reported. This underscores the importance of immune complex-mediated pathophysiology as an important role in bowel-associated dermatosis-arthritis syndrome. It suggests that properly controlling the underlying inflammatory bowel disease will play an important role in the management of IBD [7]. The American College of Gastroenterology recommended the surveillance colonoscopies in patient with UC at 1–3-year intervals based on the risk factors to develop a colorectal cancer and the significant findings on the previously done colonoscopy [8]. Arthritis may occur in 25% of patients with UC, and this finding might proceed the diagnosis of the disease [9]. Although the presentation of both conditions can be confusing, extraintestinal manifestations of IBD should be distinguished from BADAS, and the therapeutic strategies will depend entirely on the accurate diagnosis of the patient's condition. Enthesitis is seen in various rheumatic diseases, mainly in spondyloarthropathy, especially with psoriatic arthritis (PsA). Coexistence of PsA and IBD, entities known to share some of the common genetic predisposition, may increase the risk of both disorders (HLA-B27, HLA-DRB1 *∗* 0103, and non-HLA polymorphisms) [10]. The goal of treatment is to achieve low or minimal disease activity in order to control the articular (e.g., enthesitis) and extraarticular manifestations (e.g., IBD).

## 4. Conclusion

In patients with acute active arthritis, rash, and, as in this case, enthesitis with a history of gastrointestinal disease (e.g., ulcerative colitis and Crohn's disease), it is reasonable to list BADAS as a differential along with extraintestinal manifestations of inflammatory bowel disease. BADAS can recur; therefore, the early recognition of the syndrome and the proper identification and treatment of the underlying cause would result in improvement in the disease burden and the patient's quality of life. Further studies are needed to precisely understand the triggers of the occurrence of BADAS, the best diagnostic tool, as well as the optimal treatment strategy. Preventing the recurrence is crucial for such a rarely occurring disease.

## Figures and Tables

**Table 1:** Laboratory data during admission.

Laboratory	Result	Reference range
Haemoglobin	145 g/L	130–170 g/L
WBC	10.4 xE9/L	4.0–11.0 xE9/L
Platelets	497 xE9/L	150–400 xE9/L
Creatinine	99 umol/L	61–113 umol/L
ALT	36 U/L	<38 U/L
ALBUMIN	47 g/L	36.0–51.0 g/L
ANA	Negative	Negative
dsDNA Ab	4 IU/ml	<10 IU/ml
C3	1.53 g/L	0.98–1.96 g/L
C4	0.23 g/L	0.1–0.4 g/L
Rheumatoid factor	<10 IU/ml	<14 IU/ml
Anti-MPO	<0.2 AI units	≤0.9 AI units
Anti-PR3	<0.2 AI units	≤0.9 AI units
CRP	64 mg/L	0–8 mg/L
ESR	84 mm/r	0–21 mm/h
HLA-B27	Negative	Negative

Abbreviations: ANA, antinuclear antibody; dsDNA Ab, anti-double-stranded DNA antibody; C3 and C4, complement component 3 and complement component 4; Anti-MPO, anti-myeloperoxidase antibodies; Anti-PR3, anti-proteinase 3 antibody; CRP, c-reactive protein; ESR, erythrocyte sedimentation rate; HLA-B27, human leukocyte antigen B27.
